# Aquareovirus NS80 Initiates Efficient Viral Replication by Retaining Core Proteins within Replication-Associated Viral Inclusion Bodies

**DOI:** 10.1371/journal.pone.0126127

**Published:** 2015-05-04

**Authors:** Liming Yan, Jie Zhang, Hong Guo, Shicui Yan, Qingxiu Chen, Fuxian Zhang, Qin Fang

**Affiliations:** 1 State Key Laboratory of Virology, Wuhan Institute of Virology, Chinese Academy of Sciences, Wuhan 430071, China; 2 University of Chinese Academy of Sciences, Beijing, China; Institut National de la Santé et de la Recherche Médicale, FRANCE

## Abstract

Viral inclusion bodies (VIBs) are specific intracellular compartments for reoviruses replication and assembly. Aquareovirus nonstructural protein NS80 has been identified to be the major constituent for forming globular VIBs in our previous study. In this study, we investigated the role of NS80 in viral structural proteins expression and viral replication. Immunofluorescence assays showed that NS80 could retain five core proteins or inner-capsid proteins (VP1-VP4 and VP6), but not outer-capsid proteins (VP5 and VP7), within VIBs in co-transfected or infected cells. Further co-immunoprecipitation analysis confirmed that NS80 could interact with each core protein respectively. In addition, we found that newly synthesized viral RNAs co-localized with VIBs. Furthermore, time-course analysis of viral structural proteins expression showed that the expression of NS80 was detected first, followed by the detection of inner shell protein VP3, and then of other inner-capsid proteins, suggesting that VIBs were essential for the formation of viral core frame or progeny virion. Moreover, knockdown of NS80 by shRNA not only inhibited the expression of aquareovirus structural proteins, but also inhibited viral infection. These results indicated that NS80-based VIBs were formed at earlier stage of infection, and NS80 was able to coordinate the expression of viral structural proteins and viral replication.

## Introduction

The replication and assembly of many viruses take place in specific intracellular compartments called viral inclusion bodies (VIBs), virus factories or viroplasms [1,2,3,4]. These structures formed during viral infection concentrate viral proteins, virus genomes and host cellular factors required for viral replication and assembly, and coordinate the release of progeny particles [5,6,7,8,9,10].

Similar to many RNA and some DNA viruses, reoviruses form the VIBs in the cytoplasm to establish efficient genome replication and particle assembly [11,12,13]. Previous studies had demonstrated that either one or two nonstructural proteins of reoviruses are required for forming the VIBs. The nonstructural protein μNS of mammalian orthoreoviruses (MRV) and avian orthoreoviruses (ARV) formed VIBs in cells when expressed alone or during viral infection [14,15,16,17]. Similar to MRV and ARV, nonstructural protein NS2 of bluetongue virus (BTV) formed VIBs in singly expressed cells [18]. However, the VIBs formed by rotaviruses (RV) needed two nonstructural proteins, NSP2 and NSP5 [13,19].

VIBs are thought to provide a physical scaffold to concentrate viral components and related cellular factors, thereby increase the efficiency of viral replication. MRV μNS protein may retain the nonstructural protein σNS and inner-capsid proteins λ1, λ2, λ3, μ2 and σ2 within VIBs by interacting with these proteins [14,20,21,22,23]. ARV μNS protein also retained the inner-capsid proteins λA, λB and μA within VIBs [24]. In addition, RV inner-capsid proteins VP1, VP2, VP3 and VP6 were also embedded within VIBs by interacting with NSP5 [25,26]. A recent study reported that the inner-capsid proteins VP1 and VP2 of RV interacted with NSP2 [27,28]. Except for viral proteins identified in VIBs, cellular microtubule was involved in VIBs formation for most MRV strains, since microtubule-depolymerization affected the phenotype of VIBs [23,29]. Recently, another report of MRV revealed that host ribosomal subunits and proteins involved in translation were also localized within VIBs [30]. For RV, cellular lipid droplet was shown to have association with VIBs, and its disruption affected the formation of VIBs and inhibited viral replication [31]. Notably, some reports of MRV indicated that damage of VIBs by RNA interference severely restrained viral replication and progeny particle production [32,33,34].

Aquareoviruses are member of *Spinareovirinae* subfamily in *Reoviridae* and mainly infect aquatic animals [35]. GCRV-873 (Grass Carp reovirus) had been recognized as the most pathogenic amongst isolated aquareoviruses [36,37]. The genome of GCRV consists of eleven segments of double-stranded RNA (dsRNA), which encode seven structural proteins (VP1-VP7) and five nonstructural proteins (NS80, NS38, NS31, NS26 and NS16) [38]. The structural proteins assemble a double-layered (inner-capsid/core and outer-capsid) viral particle with icosahedral symmetry. Recent three-dimensional structural reconstructions by Cryo-EM indicate that inner-capsid or core proteins VP3 and VP6 build the inner shell frame. VP1 (capping enzyme), VP2 (RNA dependent RNA polymerase, RdRp) and VP4 (nucleoside triphosphatase, NTPase) proteins form the RNA polymerase complex located at five-fold axes. These core proteins have been recognized to be responsible for viral replication [39,40]. And VP5-VP7 heterohexamers are organized into viral outer-capsid, which are related to virus-cell interaction and mediate virus entry during infection [41,42]. Regarding nonstructural protein, our previous study had demonstrated that NS80 encoded by S4 segment formed VIBs when expressed alone in transfected cells or during viral infection [43,44]. The carboxyl-terminus (C-terminus) and His569 and Cys571 of NS80 were shown to be essential for VIBs formation. These findings suggested that NS80 was the minimal viral factor required for VIBs formation. Furthermore, NS80 was identified to interact with viral non-structural protein NS38 within VIBs in co-transfected and infected cells [44]. Previous study had shown that ectopically expressed core proteins VP1-VP4 and VP6 of aquareovirus co-localized with ectopic expression of NS80 in transfected cells [45]. However, the direct interactions between NS80 and inner-capsid proteins VP1-VP4 and VP6 under physiological conditions were unknown, which need intensive study. And also, compared to other reoviruses, the roles of NS80 in the aquareovirus life cycle remain poorly understood.

In this study, we firstly identified the co-localization of NS80 and inner-capsid proteins (VP1-VP4 and VP6) in co-transfected and infected cells by immunofluorescence (IF). Co-immunoprecipitation (co-IP) assay further confirmed their potential interactions, indicating that NS80 could retain inner-capsid proteins within VIBs. However, no co-localization or interaction was identified between NS80 and outer-capsid proteins (VP5 and VP7) in co-transfected or infected cells. Moreover, the synthesis of viral nascent RNAs was found to localize within VIBs by labeling newly made RNAs with bromouridine 5’-triphosphate (BrU). Time course detection of viral structural proteins expression and mRNA accumulation demonstrated that NS80 was detected prior to inner shell protein VP3 and other viral structural proteins, suggesting that NS80 related VIBs are critical to provide a platform for viral replication and virus morphogenesis. Further investigation showed that knockdown of NS80 by shRNA inhibited the expression of viral structural proteins, and also impaired virus replication and caused more than 4 log_10_ reductions of infectious yields. Taken together, these results revealed that NS80 initiates efficient viral replication by retaining inner-capsid proteins within VIBs as well as constructing its matrix.

## Material and Methods

### Cells, virus and antibodies

HEK 293T cells and Vero cells were grown in Dulbecco’s modified Eagle medium (DMEM) (Gibco-BRL) supplemented with 10% fetal bovine serum (FBS) and 100 U/ml of penicillin and streptomycin. FHM (*Fathead minnow*) cells were maintained in medium 199 (M199) (Gibco-BRL) and CIK (*Ctenopharyngodon idellus* kidney) cells were grown in minimum essential medium (MEM) (Gibco-BRL) supplemented with 10% FBS respectively. GCRV-873 isolated and stored in the author’s laboratory was propagated in CIK or FHM cells as described previously [36,46].

The VP2, VP4, VP5, VP6, VP7 and NS80 polyclonal antibodies (pAbs) of GCRV were prepared and stored in our laboratory [40,41,47,48]. VP1 and VP3 conserved regions were cloned into pRSET vector respectively based on GenBank sequences AF260511 and AF260513, and mouse polyclonal sera were prepared as described previously [48]. Mouse monoclonal antibody (mAb) against β-actin was purchased from Santa Cruz Biotechnology (Santa Cruz, CA). Horseradish peroxidase (HRP)-conjugated goat anti-rabbit IgG (H+L) or goat anti-mouse IgG (H+L) was purchased from Thermo Scientific Pierce. Alexa Fluor 488 or 568 donkey anti-rabbit IgG (H+L) antibody and Alexa Fluor 488 or 568 donkey anti-mouse IgG (H+L) antibody were purchased from Invitrogen Co. (Invitrogen, Carlsbad, USA). Mouse mAb against bromodeoxyuridine (BrdU) was the product of Sigma (Sigma-Aldrich, USA).

### Plasmids construction

Plasmids pCI-neo-VP4, pCI-neo-VP6 and pCI-neo-NS80 were prepared previously [47,48]. The VP1, VP2, VP3, VP5 or VP7 gene amplified from cDNA of GCRV based on GenBank sequence (AF260511, AF260512, AF260513, AF403392, AF403396), was cloned into pCI-neo vector (Promega, USA) and named pCI-neo-VP1, pCI-neo-VP2, pCI-neo-VP3, pCI-neo-VP5 and pCI-neo-VP7 respectively. All primers targeting structural proteins in this study were listed in S1 Table. The VP3 and VP5 gene were cloned into the *Xho* I and *Sma* I sites of the pCI-neo vector. VP1, VP2 or VP7 gene was cut by *EcoR* I /*Sal* I, *EcoR* I / *Sma* I or *EcoR* I /*Xba* I and ligated into pCI-neo vector respectively. All recombinant constructs were identified by enzyme digestion and confirmed by sequencing. All enzymes, except for T4 DNA ligase (New England BioLabs, Massachusetts), used for cloning procedures were purchased from Takara Co. (Dalian, China).

### Co-immunoprecipitation assay and Western blot analysis

Co-immunoprecipitation (co-IP) assays were performed as previously described [44]. Briefly, HEK 293T cells were co-transfected with 10 μg of each indicated plasmid. Transfected cells were harvested at 24 h post-transfection and lysed on ice with 700 μl lysis buffer. For virus infection, FHM cells were infected with GCRV and lysed on ice with 700 μl lysis buffer when the cytopathic effect (CPE) was observed. For each IP, specific polyclonal antibodies that had been incubated with 30 μl of 1:1 slurry of Protein A/G Plus-agarose (Santa Cruz, California) were added to a 0.5 ml aliquot of lysate and then rotated for at least 4 h or overnight at 4°C. The beads were washed four times with 1 ml lysis buffer and then subjected to western blot analysis. All co-IP assays were repeated three times, similar data were obtained, and a typical blot was shown.

Western blot (WB) analysis was performed as previously described [49]. Briefly, whole-cell extracts harvested at different times were subjected to 10% SDS-PAGE and transferred to polyvinylidene fluoride (PVDF) membranes, followed by blocking with 5% non-fat milk in phosphate-buffered saline containing 0.5% Tween 20 (PBST) and probed with the indicated primary antibodies at 37°C for 2 h. After washing with PBST, the membrane was incubated with HRP-conjugated secondary antibodies. The signal was detected using Super Signal West Pico Chemiluminescent substrate (Pierce, USA), imaged with the Fluochem HD2 Imaging System (Alpha Innotech, USA). Image analysis was performed with Image J software (http://rsb.info.nih.gov/nih-image).

### Immunofluorescence assay and transfection of BrU

Immunofluorescence (IF) assays were performed as described previously [47]. Briefly, Vero cells were transfected with indicated plasmids according to the user manual of Lipofectamine 2000 (Lipo 2000) (Invitrogen, Carlsbad, USA) and fixed at 24 h post-transfection. FHM cells infected with GCRV were fixed in 4% paraformaldehyde. After permeabilization by Triton X-100, all cells were incubated with the appropriate Alexa Fluor-labeled secondary antibodies following incubation with primary antibodies. After each incubation step, cells were washed extensively. Hoechst staining was applied to detect the cell nucleus. All samples were observed using Olympus-IX51 inverted microscope.

To visualize viral RNAs, FHM cells were infected and transfected with a uridine analog, bromouridine 5’-triphosphate (BrU) (Sigma-Aldrich, USA), which could be incorporated into RNA as its synthesis [50]. In detail, FHM cells were infected with GCRV for different times at MOI of 1, and then treated with 10 ug/ml of actinomycin D (ActD) (Sigma-Aldrich, USA) for 30 min to prevent cellular transcription. After treatment, cells were transfected with 10 mM BrU by using 3.7 μl Lipo 2000 dissolved in 100 μl M199 in the presence of ActD and incubated for 60 min before labeling with BrU antibody or double labeling with NS80 antibody [50].

### Real-time PCR and data analysis

The Real-time PCR (RT-PCR) primers designed by Primer Premiere software (Premier Biosoft International, Palo Alto, CA) were shown in S2 Table. All primers used in this study were determined by the slope value of the standard curve, and were shown to possess amplification efficiency greater than 90% (data not shown). In brief, RNA was extracted from 10^6^ cells by Simply P Total RNA Extraction kit (Bio Flux, China) according to the manual instruction. Quantitative PCR (qPCR) was performed on a real-time thermo cycler (Bio-Rad, USA) with the CFX 96 software. Amplification was performed by denaturing at 95°C for 3 min, followed by 40 cycles of 95°C for 15 s and 60°C for 30 s. Each reaction was performed in triplicate and β-actin was used as internal control. The RT-PCR data were analyzed by 2^−ΔΔ^ CT method [51].

Each assay was performed in three independent experiments. All data were analyzed by Statistica 6.0 (StatSoft, Inc., Tulsa, USA) and presented as the means ± standard deviation (SD). A p-value of <0.05 (*) was considered statistically significant and a p-value of <0.01 (**) was considered statistically highly significant. Graphs were generated by Graphpad (http://www.graphpad.com/). The detailed data is available as S1 Data.

### RNA interference experiments and viral infectivity assay

All primers of four shRNAs (shRNA-1, -2, -3, -4) targeting S4 were listed in Table 1 and cloned into pcDNA 6.2-GW/EmGFP-miR vectors (Invitrogen, Carlsbad, USA). CIK cells were transfected with each shRNA as described above and infected with GCRV at MOI of 1. Following incubation at 28°C for 30 min, the inoculum was removed and rinsed three times with cell culture medium, subsequently incubated in complete medium. Infected cells were collected and subjected to RT-PCR and WB analysis. Meanwhile, cell supernatant (extracellular virus) was harvested and virus titers were determined by 50% tissue culture infective dose (TCID50) assay in CIK cells. TCID50 assays were performed in 96-well cell plates (Corning, USA) with 8 replicates per dilution from 10^−1^ to 10^−8^ in MEM for 5 days. Viral infectivity was calculated by the formula of Reed and Muench [52].

**Table 1:** Oligonucleotides used to produce shRNA expression vectors.

construct	sequences of shRNA (5’-3’)
shRNA-control	**F**: TGCTG**AAATGTACTGCGCGTGGAGAC**GTTTTGGCCACTGACTGAC**GTCTCCACGCAGTACATTT**
	**R**:CCTG**AAATGTACTGCGTGGAGAC**GTCAGTCAGTGGCCAAAAC**GTCTCCACGCGCAGTACATTT**C
shRNA-1	**F**: TGCTG**ATCAGAGGAGCACCATATTCG**GTTTTGGCCACTGACTGAC**CGAATATGGCTCCTCTGAT**
	**R**: CCTG**ATCAGAGGAGCCATATTCG**GTCAGTCAGTGGCCAAAAC**CGAATATGGTGCTCCTCTGATC**
shRNA-2	**F**: TGCTG**TTCGAAGTGACGCTCCTTACC**GTTTTGGCCACTGACTGAC**GGTAAGGAGTCACTTCGAA**
	**R**: CCTG**TTCGAAGTGACTCCTTACC**GTCAGTCAGTGGCCAAAAC**GGTAAGGAGCGTCACTTCGAAC**
shRNA-3	**F**: TGCTG**TGACGTTGAAGAGCGGTGTTG**GTTTTGGCCACTGACTGAC**CAACACCGCTTCAACGTCA**
	**R**: CCTG**TGACGTTGAAGCGGTGTTG**GTCAGTCAGTGGCCAAAAC**CAACACCGCTCTTCAACGTCAC**
shRNA-4	**F**: TGCTG**TTGGGATTCAAGTTCGACGGC**GTTTTGGCCACTGACTGAC**GCCGTCGATTGAATCCCAA**
	**R**: CCTG**TTGGGATTCAATCGACGGC**GTCAGTCAGTGGCCAAAAC**GCCGTCGAACTTGAATCCCAAC**

Uppercase letters indicate oligonucleotides contained 19-mer hairpin structure. Letters in bold show sequence corresponding to S4 genome.

## Result

### Interactions between NS80 and inner-capsid proteins VP1-VP4 and VP6 in co-transfected cells

**Fig 1:**
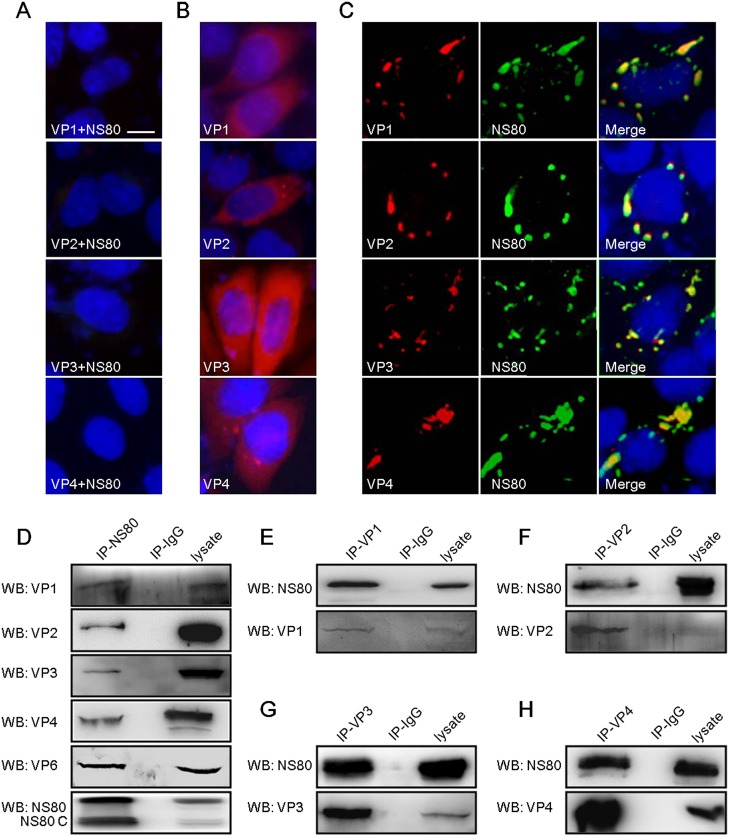
Interactions of NS80 with inner-capsid proteins (VP1-VP4 and VP6) in transfected cells. (A, B, C) IF assay of NS80 and inner-capsid proteins. Vero cells were transfected with pCI-neo (A), pCI-neo-VP1, pCI-neo-VP2, pCI-neo-VP3 or pCI-neo-VP4 plasmids (B), or co-transfected with plasmids pCI-neo-NS80 and pCI-neo-VP1, pCI-neo-VP2, pCI-neo-VP3 or pCI-neo-VP4 (C) separately. After fixation, cells were stained with mouse polyclonal antibodies against VP1, VP2, VP3 or VP4 (B), and with rabbit polyclonal antibody against NS80 (A and C) respectively. Cell nuclei were counterstained with Hoechst. Scale bars, 10 μm. (D-H) Co-IP assays of NS80 and inner-capsid proteins. HEK 293T cells were co-transfected with plasmids expressing NS80 and VP1 (E), VP2 (F), VP3 (G), VP4 (H) or VP6 respectively. Cells were lysed and IP assays were performed with polyclonal antibodies against NS80 (IP-NS80, D), VP1 (IP-VP1, E), VP2 (IP-VP2, F), VP3 (IP-VP3, G), VP4 (IP-VP4, H) or negative control serum (IP-IgG) respectively. IP samples were further analyzed by WB.

Our previous studies demonstrated that aquareovirus NS80 formed VIBs in singly transfected cells, and also interacted with nonstructural protein NS38 in these structures in co-transfected cells [44]. Whether inner-capsid proteins of aquareovirus also interact with NS80 within VIBs is unclear. To investigate this issue, IF assays were carried out to examine the distribution of VP1-VP4 expressed solely or co-expressed with NS80 in Vero cells using prepared specific antibodies, respectively. It appeared that the distributions of VP1, VP2, VP3 and VP4 in NS80 co-transfected cells were clearly different from that of these proteins expressed solely in Vero cells (Fig 1B and 1C). As shown in Fig 1C, the VP1, VP2, VP3 and VP4 co-localized with NS80 in co-transfected cells, respectively. No stained nonspecific immunofluorescence background was detected in mock transfected cells (Fig 1A). Based on these results, and combining the co-localization of VP6 and NS80 in co-transfected cells in our previous report [47], we could conclude that NS80 may mediate the reorganization of inner-capsid proteins (VP1-VP4 and VP6) in co-transfected cells.

In order to clarify the molecular mechanism of NS80 retaining the inner-capsid proteins within VIBs, the potential interactions between NS80 and inner-capsid proteins (VP1-VP4 and VP6) were investigated. Plasmid pCI-neo-NS80 either with pCI-neo-VP1, pCI-neo-VP2, pCI-neo-VP3, pCI-neo-VP4 or pCI-neo-VP6 was co-transfected into HEK 293T cells, respectively. And co-IP/WB analysis was performed with antibodies against NS80 and VP1-VP4 and VP6. As shown in Fig 1D, inner capsid proteins VP1-VP4 and VP6 were efficiently co-IPed with NS80 by anti-NS80 pAbs. Moreover, full-length NS80 was efficiently co-IPed with VP1, VP2, VP3 or VP4 by respective specific antibody (Fig 1E, 1F, 1G and 1H). In addition, a band of expected size (about 80 kDa) and another band with shorter size (about 70 kDa, named as NS80 C) were detected in cell lysates and co-precipitated proteins with NS80 pAbs (Fig 1D), which is corresponding to our previous report [43,44]. Taken together, these results demonstrated that NS80 interacted with five inner-capsid proteins in co-transfected cells.

### Inner-capsid proteins VP1-VP4 and VP6 are localized within VIBs in aquareovirus infected cells

**Fig 2:**
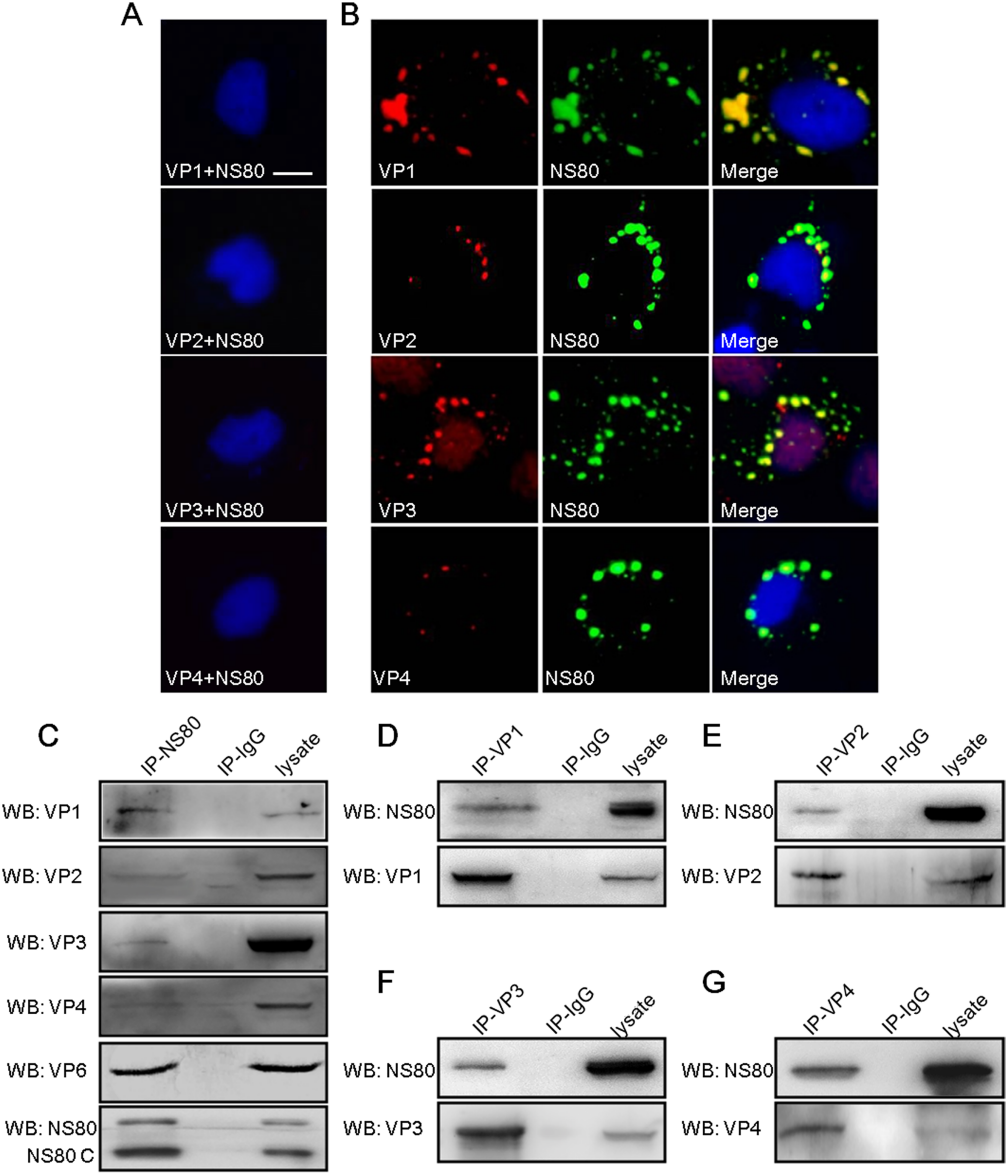
Interaction of NS80 with inner-capsid proteins (VP1-VP4 and VP6) in infected cells. (A, B) Colocalization of NS80 and proteins VP1-VP4 in infected cells. Mock infected (A) or GCRV infected FHM cells (B) were fixed and stained with mouse polyclonal antibodies against VP1, VP2, VP3 or VP4 and rabbit polyclonal antibody against NS80 respectively. Cell nuclei were counterstained with Hoechst. Scale bars, 10 μm. (C-G) Co-IP assays of NS80 and inner-capsid proteins. FHM cells infected with GCRV were lysed and IP assays were performed with polyclonal antibodies against NS80 (IP-NS80, C), VP1 (IP-VP1, D), VP2 (IP-VP2, E), VP3 (IP-VP3, F), VP4 (IP-VP4, G) or negative control serum (IP-IgG) respectively. IP samples were further analyzed by WB.

The aforementioned results showed that NS80 interacted with five inner-capsid proteins in co-transfected cells. So we hypothesized that the inner-capsid proteins might be localized within VIBs in infected cells. To test this hypothesis, IF assays were performed in mock infected or aquareovirus infected FHM cells. In agreement with Fig 1, NS80 co-localized with the inner-capsid proteins VP1-VP4 in aquareovirus infected cells (Fig 2B), and no stained nonspecific immunofluorescence background was detected in mock infected cells (Fig 2A). Next, we further investigated the interaction between NS80 and each inner-capsid proteins during infection, respectively. FHM cells infected with GCRV were lysed and co-IP/WB was performed as described in materials and methods. The results showed that VP1-VP4 and VP6 proteins were efficiently co-IPed with NS80 by anti-NS80 pAbs (Fig 2C) respectively, but not by negative control serum (IP-IgG). Besides, the interactions were verified by reciprocal co-IP assays with VP1-VP4 specific antibodies and WB analysis using NS80 antibody. As shown in Fig 2D, 2E, 2F and 2G, NS80 was efficiently co-IPed with VP1, VP2, VP3 or VP4 by respective specific antibody. Consistent with above results of transfected cells, two forms of NS80 (NS80 and NS80 C) were also detected in infected cell lysates and co-precipitated proteins with NS80 pAbs (Fig 2C), while only full-length NS80 was observed in co-precipitated proteins with inner-capsid proteins pAbs (Fig 2D, 2E, 2F and 2G), suggesting that the interaction between full-length NS80 and inner-capsid proteins might be more stable than that of NS80 C. Taken together, these results demonstrated that aquareovirus inner-capsid proteins (VP1-VP4 and VP6) were retained within VIBs by interacting with NS80 during infection.

### Outer-capsid proteins VP5 and VP7 are not localized within VIBs

**Fig 3:**
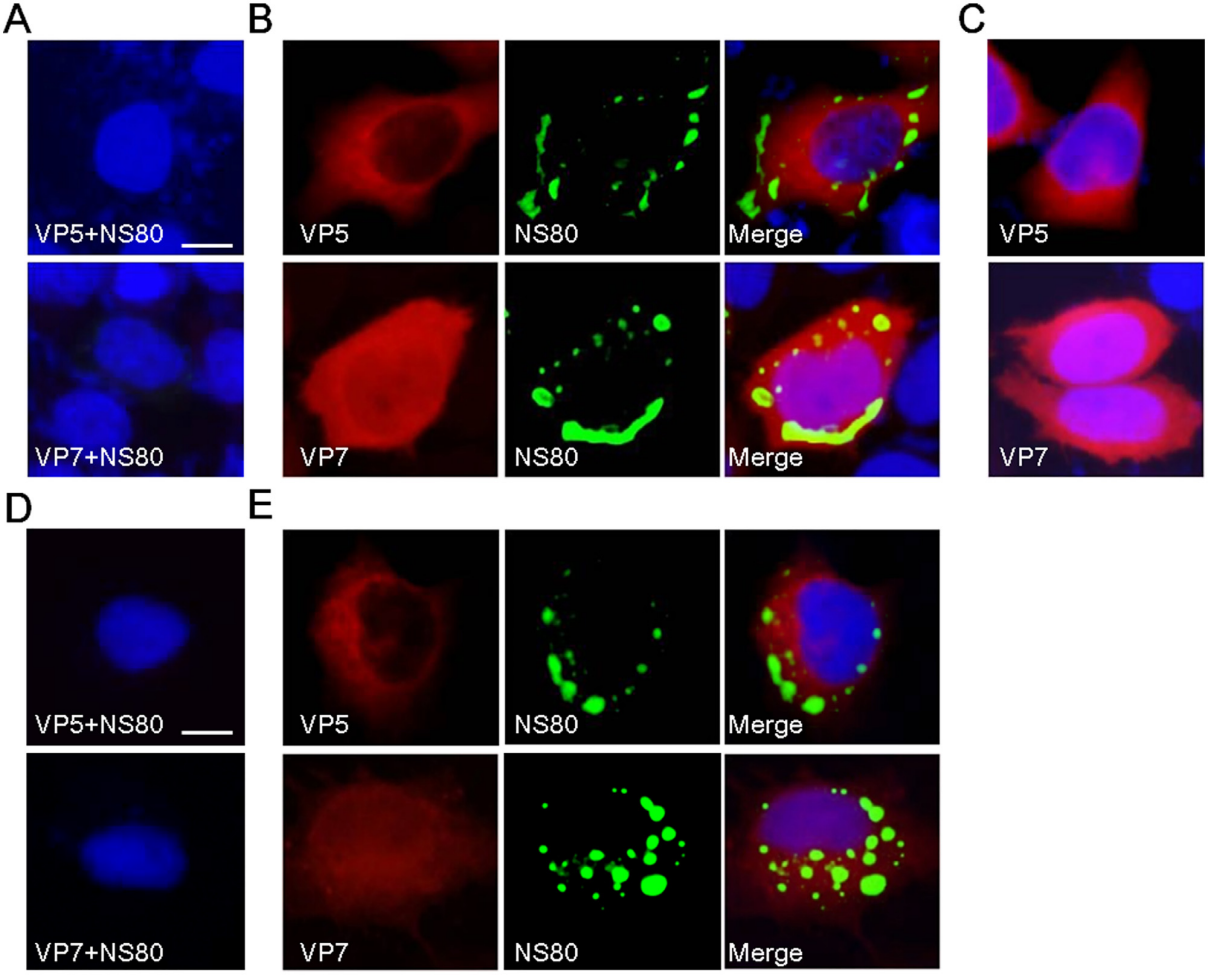
NS80 did not colocalize with outer-capsid proteins VP5 and VP7 in transfected or infected cells. (A, B, C) IF assay of NS80 and outer-capsid proteins in co-transfected cells. Vero cells were transfected with pCI-neo (A), pCI-neo-VP5 or pCI-neo-VP7 (C), or co-transfected with plasmids pCI-neo-NS80 and pCI-neo-VP5 or pCI-neo-VP7 (B) separately. (D, E) IF assay of NS80 and outer-capsid proteins in infected cells. Mock infected (D) or GCRV infected FHM cells (E) were fixed. After fixation, all cells were stained with mouse polyclonal antibodies against VP5 or VP7 (A-E), and with rabbit polyclonal antibody against NS80 (A, B, D and E). Cell nuclei were counterstained with Hoechst. Scale bars, 10 μm.

The inner-capsid proteins were localized within VIBs during infection, suggesting that the viral cores are replicated and assembled within VIBs. However, the place where viral cores were coated by outer-capsid proteins is still unknown. To determine whether outer-capsid proteins VP5 and VP7 are also associated with NS80, IF was used to investigate the distribution of VP5 and VP7 expressed alone or co-expressed with NS80 in Vero cells, respectively. As shown in our results, the diffusely distributed pattern of VP5 and VP7 in NS80 co-transfected cells were the same as these proteins expressed alone in Vero cells (Fig 3B and 3C). And neither VP5 nor VP7 was co-localized with NS80 in co-transfected cells (Fig 3B). In accordance with the results from co-transfected cells, the outer-capsid proteins VP5 and VP7 were not co-localized with NS80 in aquareovirus infected cells (Fig 3E). No stained nonspecific immunofluorescence background was detected in mock transfected and infected cells (Fig 3A and 3D). Further co-IP assay indicated that no interactions were detected between NS80 and VP5 or VP7 in co-transfected cells or infected cells (data not shown). These results suggested that outer-capsid proteins VP5 and VP7 were not localized within VIBs.

### Newly synthesized aquareovirus RNA is localized to VIBs in infected cells

**Fig 4:**
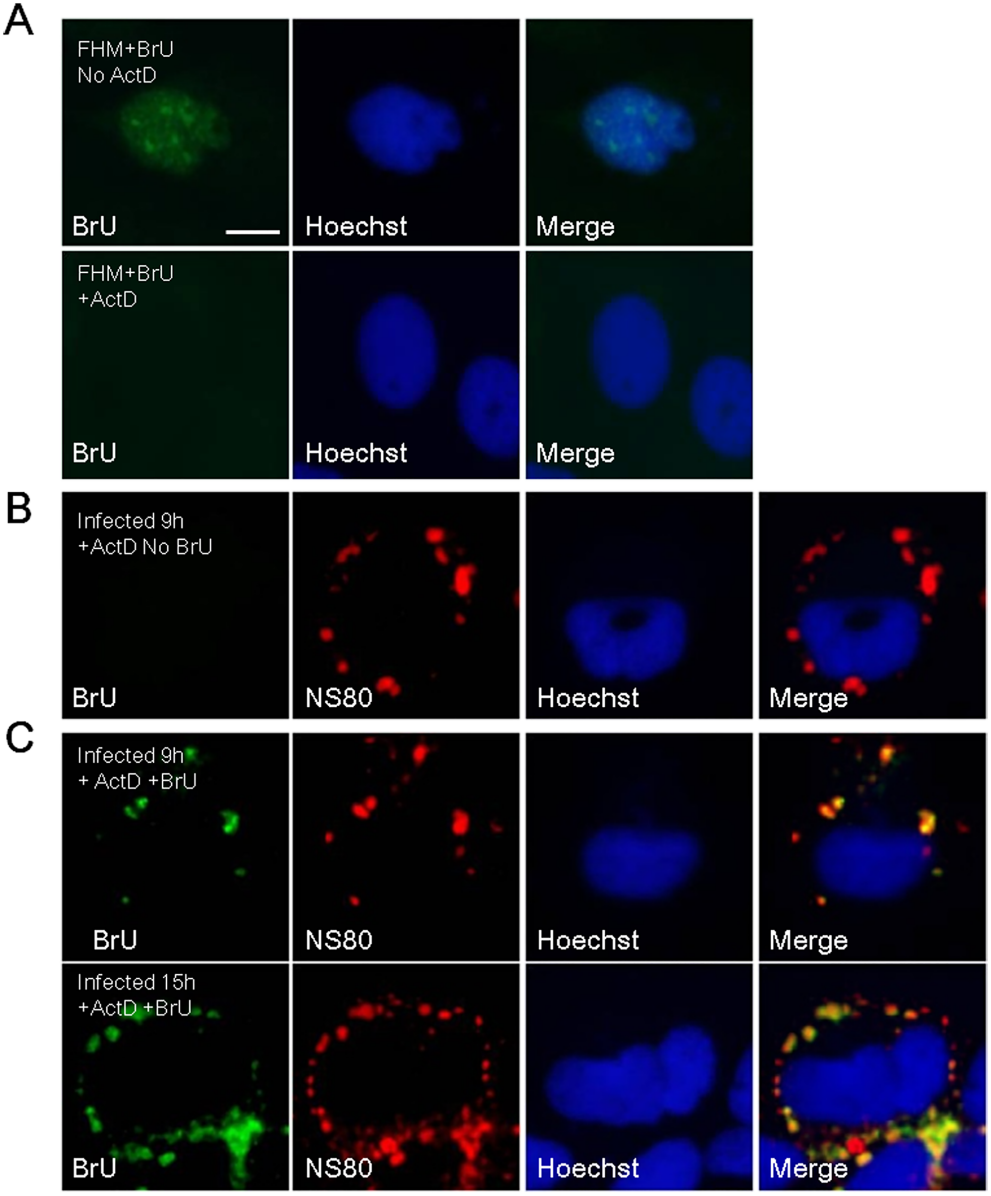
Localization of newly synthesized RNAs to VIBs. (A) FHM cells were transfected with BrU in treatment with or without ActD and stained with mouse monoclonal antibody against BrU. (B) FHM cells infected with GCRV at MOI of 1 were transfected without BrU (No BrU) in the presence of ActD for 1 h at 9 h post-infection. (C) FHM cells infected with GCRV at MOI of 1 were transfected with BrU (+BrU) in the presence of ActD for 1 h at 9 h or 15 h post-infection. Infected cells were stained with mouse monoclonal antibody against BrU and rabbit polyclonal antibody against NS80. Cell nuclei were counterstained with Hoechst. Scale bars, 10 μm.

Our aforementioned results showed that viral inner-capsid proteins including RNA synthesis related proteins VP2 and VP4 localized to VIBs formed by NS80, which suggested that viral transcriptase core particles are embedded in VIBs in infected cells. In this regard, we hypothesized that VIBs might be the site of viral RNA synthesis. To test this issue, FHM cells infected with GCRV were treated with BrU following treatment with ActD to prevent cellular transcription. After fixation, VIBs were visualized by NS80 polyclonal antibody and newly made RNA was visualized by BrdU antibody, which cross reacted with BrU. As shown in Fig 4C, BrU-labeled viral plus-strand RNA was distributed in cytoplasmic region with a discrete phenotype, and localized within VIBs formed by NS80 in infected cells, supporting our hypothesis that viral RNAs synthesized by transcriptionally active core particle occurred within VIBs. However, no stained BrU was detected in the absence of BrU treatment (Fig 4B), indicating no cross-reaction happened between NS80 and BrU antibodies. Moreover, newly transcribed RNA was not observed in FHM cells treated with ActD, and an alternative pattern of nuclear staining was observed without ActD treatment (Fig 4A). Taken together, these results demonstrated that newly synthesized viral RNA is localized to VIBs in infected cells.

### The expression of NS80 is detected ahead of viral structural proteins

**Fig 5:**
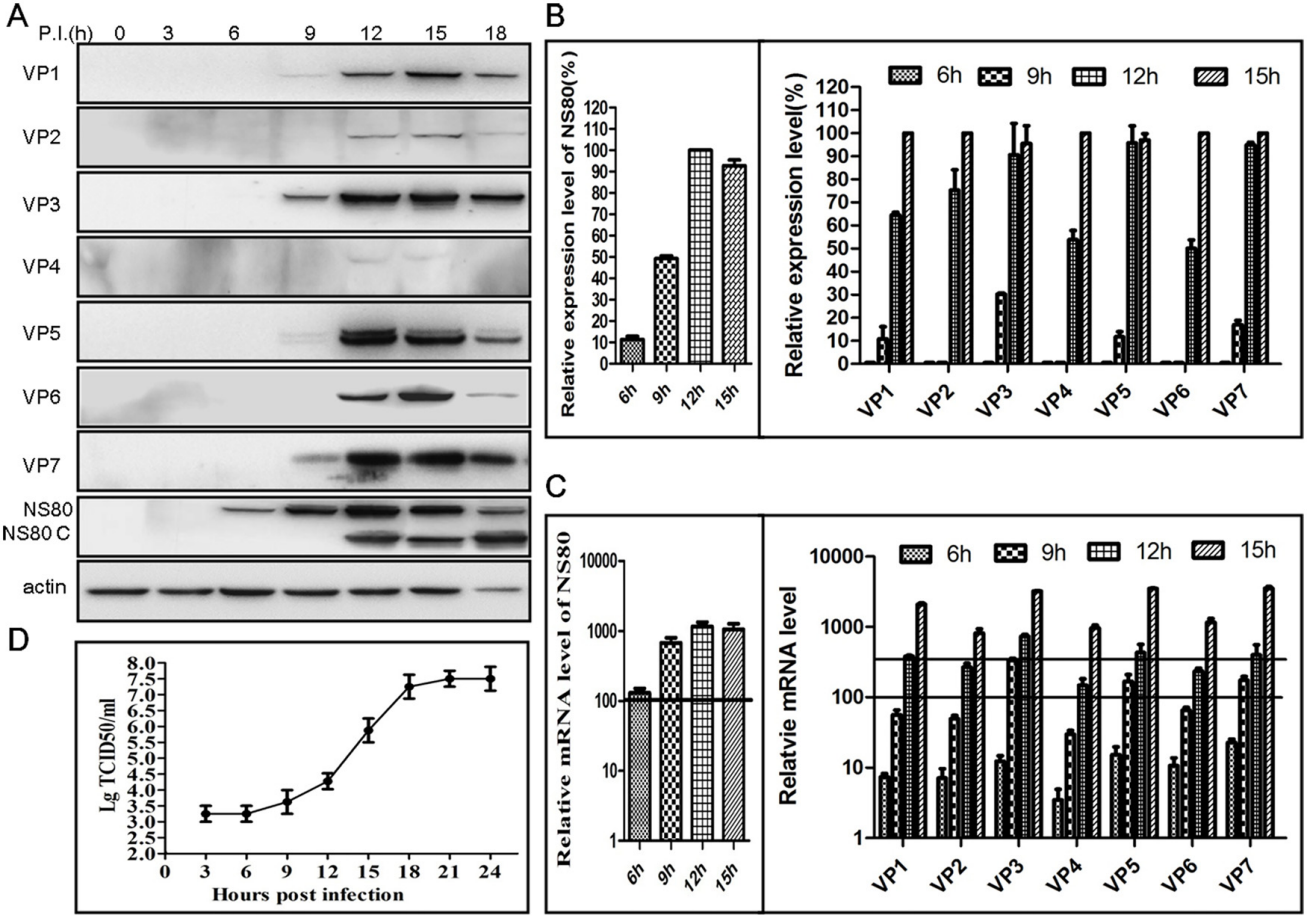
Time-course detection of viral proteins expression and mRNA accumulation during viral replication cycle. (A, B, C) WB and quantitative RT-PCR assays of viral proteins expression. FHM cells infected with GCRV at MOI of 1 were collected at indicated times and analyzed by WB to detect the expression of all structural proteins and NS80. One of three independent WB results was shown in (A). Meanwhile, protein relative expression level was balanced to β-actin and then evaluated to maximum value. Results of three independent experiments were shown in (B). Infected FHM cells were collected at indicated times post-infection and assessed by RT-PCR. All mRNA relative level was balanced to β-actin and evaluated to samples of 3 h post-infection and shown in (C). (D) One-step growth curve of GCRV. The data represent means plus standard deviations for three independent experiments.

Aquareovirus NS80 could form the VIBs during infection, and the above results showed that newly synthesized viral plus-strand RNA is also localized to VIBs. In order to further determine the role of VIBs in viral replication, we detected the expression of NS80 and viral structural proteins at different time point. FHM cells were infected with aquareovirus at MOI of 1, and then the cells and supernatant were collected at 3, 6, 9, 12, 15, and 18 h p.i.. The infected cells were subjected to WB or RT-PCR, and culture supernatants were analyzed by TCID50. As shown in our results, the expression of NS80 protein was firstly detected at 6 h p.i. (Fig 5A), and the proportion of expression was about 12% (Fig 5B). Meanwhile, the mRNA of NS80 was detected by quantitative RT-PCR, and level of NS80 mRNA was about 100 fold increases (Fig 5C). The expression of inner shell VP3 protein was followed the NS80 and could be detected at 9 h p.i. (Fig 5A), and the proportion of VP3 expression was about 30% (Fig 5B). Inner turret VP1 protein and outer-capsid proteins (VP5 and VP7) could also be detected synchronously, while their value was clearly lower than VP3 protein, which were further confirmed by quantitative RT-PCR (Fig 5B and 5C). This result suggested that VP3 might be firstly expressed prior to other viral structural proteins. Moreover, the detection of inner clamp VP6, minor proteins VP2 and VP4 was following NS80 and VP3 at 12 h p.i. (Fig 5A, 5B and 5C). At the same time, viral growth curve was generated by TCID50 assays. As shown in Fig 5D, the virus titers remained no difference from 3 h to 6 h p.i.. While, virus titers gradually increased from 6 h to 12 h p.i., which was in accord with the detection of viral structural proteins. Along with the expression of NS80 and viral structural proteins, the virus titers could go up to highest at 18 h p.i.. Taken together, these results indicated that the expression of NS80 protein was detected ahead of viral structural proteins, suggesting that NS80 may play very important role in viral replication, and also VIBs may provide a platform for viral replication and assembly to facilitate viral infection process.

### Knockdown of NS80 inhibits the expression of viral structural proteins and viral replication

**Fig 6:**
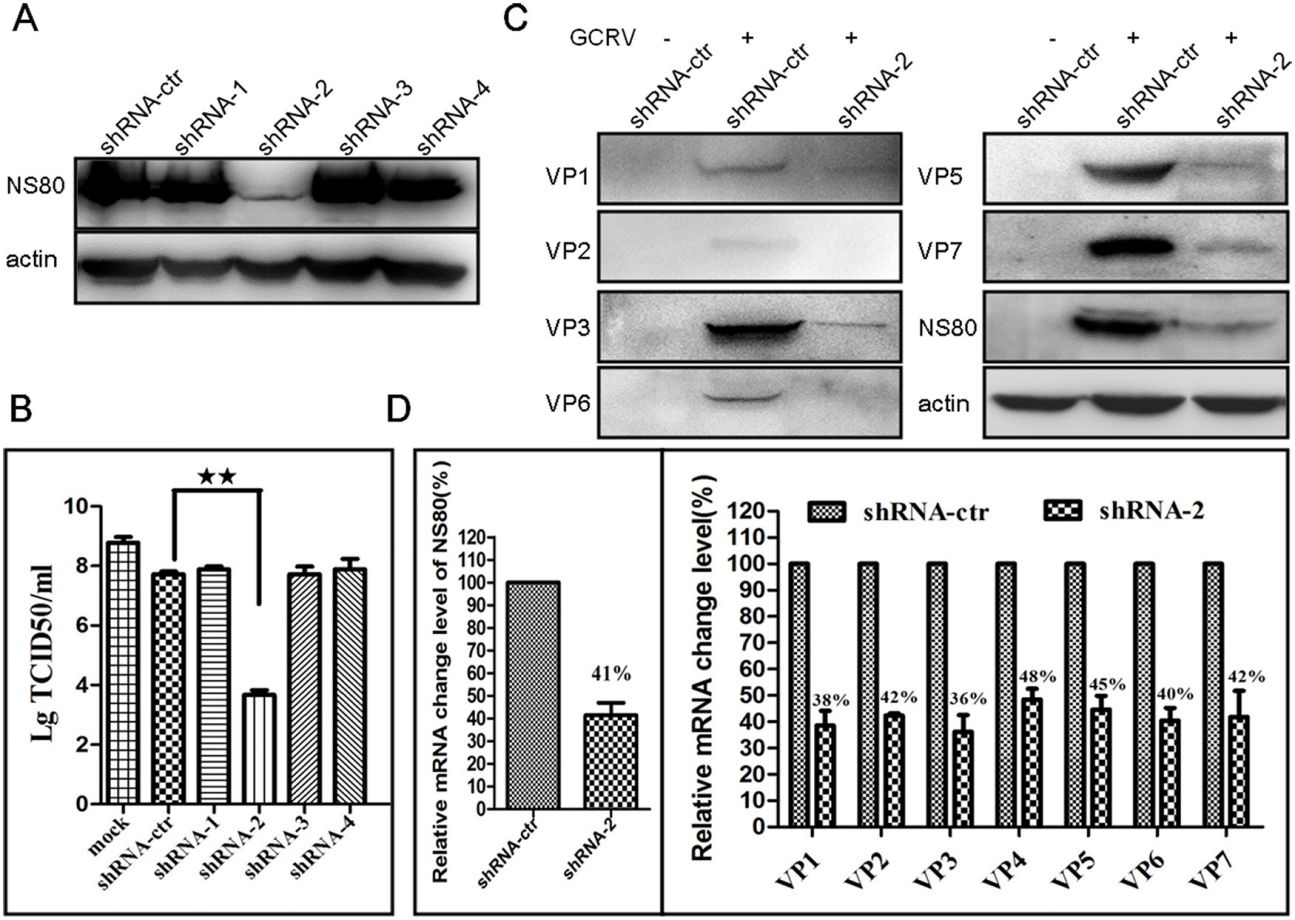
Knockdown of NS80 impaired virus replication. (A) HEK 293T cells were co-transfected with each shRNA construct and pCI-neo-NS80. All the treated cells were analyzed by WB at 24 h post-transfection. (B) CIK cells transfected without (mock) or with each shRNA construct were infected with GCRV at MOI of 1 at 24 h post-transfection. Cell supernatants were collected at 24 h post-infection and virus titers were tested by TCID50 assay. (C) CIK cells transfected with shRNA-control (shRNA-ctr) or shRNA-2(shRNA-2) were mock infected (-) or infected with GCRV (+) at MOI of 1 at 24 h post-transfection and then analyzed by WB or RT-PCR at 24 h post-infection. (D) Relative mRNA change level in NS80 knockdown cells. All relative mRNA level in shRNA-2 transefcted cells was balanced to β-actin and evaluated to samples of shRNA-control transfected cells. The data represent means plus standard deviations for three independent experiments. Statistical analysis was performed using Student’s *t* test. ** indicates *P*<0.01.

Our results indicated that the expression of aquareovirus NS80 was detected at 6 h p.i., which was ahead of viral structural proteins. So, we hypothesized that the knockdown of NS80 may inhibit the expression of viral structural proteins, and thus restrict viral infection. To confirm this hypothesis, we constructed four shRNAs (shRNA-1, -2, -3, -4) targeting different regions of NS80 coding sequence (Table 1). The inhibitory efficiency of each shRNA was firstly investigated by co-transfection with pCI-neo-NS80 into HEK 293T cells, respectively. As shown in Fig 6A, the expression of NS80 was sharply decreased by shRNA-2, but not influenced by other three shRNAs. Meanwhile, the effect of the four shRNAs on viral infection was investigated. CIK cells were transfected with each shRNA and then infected with GCRV at MOI of 1 at 24 h post-transfection. Cell supernatants were harvested for virus titer assay. As shown in Fig 6B, the virus titer was about 10-fold reduction in shRNA-control transfected cells compared to mock transfected cells. In comparison to shRNA-control, the virus titer was shown to be reduced more than 4 log_10_ by transfection with shRNA-2, but nearly not influenced by other three shRNAs. These results indicated that shRNA-2 was the most efficient to inhibit NS80 expression and thus restrain viral infection. So shRNA-2 was chosen for further research.

To further investigate the effects of NS80 knockdown on the expression of viral structural proteins, the whole-cell lysates from shRNA transfected and GCRV infected cells were examined by WB and RT-PCR. The results showed that knockdown of NS80 by shRNA-2 not only inhibited the expression of viral inner-capsid proteins (VP1, VP2, VP3 and VP6), but also inhibited the expression of outer-capsid proteins (VP5 and VP7) (Fig 6C). Correspondingly, following the reduction of NS80 in mRNA level (reduced to 41%), all structural proteins in mRNA level decreased by more than half level (Fig 6D). Taken together, these results demonstrated that knockdown of NS80 may damage VIBs formation and thus restrain viral replication and progeny virus production, suggesting that NS80 plays crucial role in viral structural proteins expression and viral replication.

## Discussion

Previous studies demonstrated that MRV, ARV and RV generated VIBs in cytoplasm during infection, and it has been recognized that the VIBs contained newly synthesized viral structural proteins, RNA and nascent viral particles [5,6,7,8,9,10]. Aquareovirus also formed VIBs in cytoplasm of infected cells, and NS80 protein was identified to induce the formation of VIBs in our previous report [43,44]. In this study, we verified that NS80 could retain the inner-capsid proteins (VP1-VP4 and VP6), but not outer-capsid proteins (VP5 and VP7), within VIBs by direct interactions. And also, we indicated that NS80 plays critical role in the expression of viral structural proteins and viral replication.

Aquareovirus particles are composed of seven structural proteins, including inner-capsid proteins (VP1-VP4 and VP6) and outer-capsid proteins (VP5 and VP7). Previous report had shown that FLAG-NS80 co-localized with VP1-HA, VP2-HA, VP3-HA, VP4-HA in transfected cells [45], but the direct interactions between NS80 and inner-capsid proteins (VP1-VP4 and VP6) under physiological conditions needed to be clarified. In this study, we demonstrated that NS80 altered the distribution of singly expressed inner-capsid proteins (VP1-VP4 and VP6) and retained them within VIBs in co-transfected cells by using our prepared specific antibodies. We also found that inner-capsid proteins (VP1-VP4 and VP6) were localized within VIBs formed by NS80 in infected cells. And next, co-IP analysis was employed to further determine the interaction between NS80 and inner-capsid proteins under physiological conditions. As expected, NS80 interacted with each inner-capsid protein during infection. These results were consistent with the findings of μNS from MRV and NSP5 from RV, which also retained the inner-capsid proteins within VIBs [14,20,21,23,25,26]. However, the ARV μNS did not retain σA (the analogue of MRV σ2) within VIBs, which was the equivalent of aquareovirus VP6 [24,47]. The similar results between aquareovirus and other reoviruses suggested that NS80 was involved in retaining viral replication-related proteins for progeny virus particle assembly as well as constructing VIBs. Different from the inner-capsid proteins, the outer-capsid proteins VP5 and VP7 did not co-localize with NS80 in co-transfected or infected cells, suggesting that NS80 could not retain the outer-capsid proteins within VIBs. This result was different from the report of Rice dwarf virus (RDV) in phytoreovirus. The accumulation of outer capsid proteins (P2, P8 and P9) and intact virus particles of RDV were identified in the peripheral regions of VIBs [53]. Three-dimensional image of GCRV from cryo-EM reconstructions indicated that the outer capsid is composed of 200 trimers of VP5-VP7 heterodimers [54], and we found that the outer-capsid proteins VP5 and VP7 were capable of assembling into heteropolymer when expressed in insect cells in our recent study (Yan et al., 2015, in press), indicating that VP5 and VP7 or the homologous proteins in the *Spinareovirinae* subfamily of *Reoviridae* have to form heteropolymer complex before coating onto nascent core structure during virus replication cycle. However, the accurate site where the outer-capsid proteins were expressed and assembled onto progeny core needs to be further investigated. It may need to note that the isoform of NS80 (NS80 C) was detected in both of infected and transfected cells in this and previous study [43,44]. Other reports revealed that a similar short peptide μNS C of MRV or ARV was also found in infected cells [17,55]. These findings suggest that NS80 and its analogue proteins have another form in infected cells and they may function at different stages in regulating viral replication and assembly.

Previous studies indicated that the MRV cores serve as transcriptase particle in vivo and transcriptionally active in vitro for the production of plus-strand RNAs [56,57,58,59]. In this study, based on the fact that aquareovirus inner-capsid proteins were retained within VIBs during infection, it would be suggested that newly synthesized viral RNAs are located within VIBs. As expected, the aquareovirus plus-strand RNAs were found to localize to VIBs at very early times of infection, indicating that inner-capsid proteins are retained within VIBs as they are formed at early times in infected cells. Our previous study showed the interaction between NS80 and nonstructural proteins NS38 within VIBs. Since NS38 is homologous of MRV σNS, which was capable of binding single-stranded RNA (ssRNA), NS38 was supposed to possess ssRNA-binding activity [44,60,61]. The localization of inner-capsid proteins to VIBs may be a particularly efficient way for aquareovirus to ensure that newly transcribed viral plus-strand RNAs are retained close to the proteins necessary for replication and core assembly. In MRV, BTV, RV and phytoreovirus, the localization of nascent RNAs was also identified within VIBs in infected cells [53,59,62,63]. This suggests that diverse members of the family *Reoviridae* are similar in this regard.

The relationship between NS80 and viral structural proteins is quite complex. In this study, the expression of NS80 and viral structural proteins are detected in aquareovirus infected cells. WB results revealed that NS80 protein was detected at 6 h p.i.. And the expression of inner shell protein VP3 and inner turret VP1 protein could be detected at 9 h p.i.. However, the amount of VP3 expression was significantly higher than that of VP1 protein, maybe the expression amounts of the two proteins are related to their protein copy numbers assembled in viral particle. And also, the quantitative RT-PCR results were consistent with the WB results. Our findings that VP3 is the first detectable inner-capsid protein expressed in viral replication, and the fact that NS80 mediates the concentration of VP3 within VIBs suggest that the first step in aquareovirus morphogenesis is the formation of inner shell frame structure. In this way, the VP3 protein of aquareovirus is similar with ARV λA, which was the structural protein first expressed and retained within VIBs [24]. Subsequently, core assembly is completed by the packaging of other four viral inner-capsid proteins. At last, the cores depart from VIBs and are coated by outer-capsid proteins VP5 and VP7 to terminate enzymatic activity.

To address the role of NS80 in the expression of viral structural proteins and viral replication, RNA interference was used to knockdown the NS80 expression. In this study, we firstly confirmed that shRNA-2 was the most efficient to knockdown NS80 expression among the four tested shRNAs. Then we showed that inhibition of the virus titers with shRNA-2 was nearly 4 log_10_. Moreover, the knockdown of NS80 also restrained the expression of viral inner-capsid proteins and outer-capsid proteins, which was further confirmed in mRNA level by RT-PCR. The reduction of viral structural proteins expression suggested that most of viral proteins detected in infected cells were products of secondary transcription initiated by progeny core particles, which was in agreement with previous reports of reovirus [64,65]. These results together with kinetics of viral proteins expression strongly indicated that NS80-based VIBs were formed at the earlier stage of infection to initiate viral replication by retaining core proteins within replication-associated VIBs. These results were similar to that of other reoviruses. For example, knockdown of NSP5 from RV or μNS from MRV reduced production of infectious virus, as well as decreasing synthesis of viral mRNA and the expression of all viral structural proteins [32,33,34,66].

In summary, this is the first report to describe the role of aquareovirus NS80 in viral replication and infection. NS80 retains the inner-capsid proteins (VP1-VP4 and VP6) within VIBs, and newly synthesized viral RNAs are also co-localized with VIBs. Knockdown of NS80 by shRNA not only inhibited the expression of viral structural proteins, but also significantly restrained aquareovirus replication. The results reported in this study will lay a foundation for further elucidating the role of NS80 in aquareovirus infection and pathogenesis.

## Supporting Information

S1 Table Primers used in plasmids construct. (DOC)

S2 Table Primers used in RT-PCR. (DOC)

S1 Data The detailed data of immunofluorescence assay for negative control and relative protein expression level analysis results. (RAR)
