# Supplementary material for: Aquareovirus NS80 Initiates Efficient Viral Replication by Retaining Core Proteins within Replication-Associated Viral Inclusion Bodies
Source: PLoS One. 2015 May 4;10(5):e0126127. doi: 10.1371/journal.pone.0126127 (PMC4418822; doi:10.1371/journal.pone.0126127)
Supplement: S1 Table — (DOC) [file pone.0126127.s001.doc]

**S1 Table P**rimers used in plasmids construct

| Plasmids | Primers(5,-3,) |
| --- | --- |
| pCI-neo-VP1 | F: CTAGAATTCTTGCATTATGGCTGCGG  R: TACGTCGACAGGTGGGCGCTCGTTACTC |
| pCI- neo-VP2 | F: CATGAATTCTTGTACCATGGAGGAATTGTTCAACGCC  R: GCTCCCGGGCTAAACATCACGCATCC |
| pCI- neo-VP3 | F:CATCTCGAGATTTCCACCATGCCGCGCCGATCAGCC  R: GCTCCCGGGCGATTACGTCGCGCTGCGCAC |
| pCI- neo-VP5 | F: CATCTCGAGTCTCTACAATGGGGAACGTTCAAACC  R: GCTCCCGGGTCACTTGCCGGGCCACAAG |
| pCI- neo-VP7 | F: CATGAATTCATCACCACGATGCCACTTCAC  R: GCTTCTAGATGCTTAATCGGATGGC |
