# Supplementary material for: Aquareovirus NS80 Initiates Efficient Viral Replication by Retaining Core Proteins within Replication-Associated Viral Inclusion Bodies
Source: PLoS One. 2015 May 4;10(5):e0126127. doi: 10.1371/journal.pone.0126127 (PMC4418822; doi:10.1371/journal.pone.0126127)
Supplement: S2 Table — (DOC) [file pone.0126127.s002.doc]

**S2 Table P**rimers used in RT-PCR

| Target | *Sequence(5,-3,)* | *Product Size* |
| --- | --- | --- |
| Actin | F: ATCGTGCGTGACATTAAGGAG  R: GGAAGGAAGGCTGGAAGAG | 135 bp |
| NS80 | F: GGAAGCCGACAAGGGAATG  R: TGGAGTAGCCGTGGGAAG | 188 bp |
| VP1 | F: TACCAACCCGTTAGTGCTT  R: GGAGTAGTAGAATACCGTGGC | 289 bp |
| VP2 | F: TACGCCTACACCTTACTTCAA  R: CGGTTCGGTCCACTCTATT | 114 bp |
| VP3 | F: GCTTTCTTCATCCGAGTGG  R: GCGACGAGGACATTGGTA | 242 bp |
| VP4 | F: TGGCTCTATTGATGTCTGATG  R: CAGTGATGTGGACGAAAGG | 102 bp |
| VP5 | F: CGCCATCAATCTCGCTATCA  R: CGCCCTTGTATGTCGTCTCA | 227 bp |
| VP6 | F: CCCTGACTGGACGCCTAA  R: CGCCTGCCACTTCTACGA | 198 bp |
| VP7 | F: AGCCATTCGCTCATTAGTCG  R: TTTGGTGGGATGCTCGTTAG | 292 bp |
